# ^18^F-PSMA PET/CT Versus ^18^F-NaF PET/CT for Staging and Treating Newly Diagnosed High-Risk Prostate Cancer: A Prospective Single-Center Study

**DOI:** 10.2967/jnumed.125.270822

**Published:** 2025-12

**Authors:** Claus Madsen, Helle D. Zacho, Dan Fuglø, Kayalvily Nielsen, Per Kongsted, Rasmus Bisbjerg, Maria Pedersen, Rikke Broholm, Christian Haarmark, Peter B. Østergren

**Affiliations:** 1 Department of Nuclear Medicine, Copenhagen University Hospital Herlev and Gentofte, Herlev, Denmark;; 2 Department of Nuclear Medicine and Clinical Cancer Research Centre, Aalborg University Hospital, Aalborg, Denmark;; 3 Department of Clinical Medicine, Aalborg University, Aalborg, Denmark;; 4 Department of Radiology, Copenhagen University Hospital Herlev and Gentofte, Herlev, Denmark;; 5 Department of Clinical Medicine, Copenhagen University, Copenhagen, Denmark;; 6 Department of Oncology, Copenhagen University Hospital Herlev and Gentofte, Herlev, Denmark; and; 7 Department of Urology, Copenhagen University Hospital Herlev and Gentofte, Herlev, Denmark

**Keywords:** ^18^F-NaF PET/CT, ^18^F-PSMA PET/CT, clinical impact, prostate cancer, staging, treatment

## Abstract

Radiolabeled prostate-specific membrane antigen (PSMA) PET/CT has demonstrated superior diagnostic accuracy for staging prostate cancer (PC) compared with conventional imaging modalities. However, the clinical impact of replacing ^18^F-NaF PET/CT with PSMA PET/CT in high-risk PC remains underexplored. **Methods:** In this prospective single-center study, 160 patients with newly diagnosed high-risk PC underwent both ^18^F-NaF PET/CT and ^18^F-PSMA-1007 PET/CT within 3 wk. The ^18^F-PSMA PET/CT results were initially withheld; therefore, staging and treatment decisions were based solely on ^18^F-NaF PET/CT. At a later time point, the ^18^F-PSMA PET/CT findings were revealed, and staging and treatment plans were reassessed in a multidisciplinary research setting. Differences in staging and treatment intent were analyzed using Wilcoxon signed-rank tests. **Results:** The metastatic stage was reclassified for 40 patients (25%) after ^18^F-PSMA PET/CT, with 24% classified as having a more advanced stage and 1% having a less advanced stage (*P* < 0.001). Lymph node metastases accounted for most changes: 30 patients (19%) with no nodal involvement on CT were found to have nodal disease on ^18^F-PSMA PET/CT, and the rate of extrapelvic lymph node metastases increased from 16% to 27%. Treatment plans were changed for 21 patients (13%), primarily reflecting a shift from curative to noncurative intent or toward more intensified systemic therapy (*P* = 0.001). **Conclusion:** The use of ^18^F-PSMA PET/CT resulted in significant metastatic stage migration and influenced treatment planning in a substantial proportion of patients.

## Visual Abstract

**Figure:**
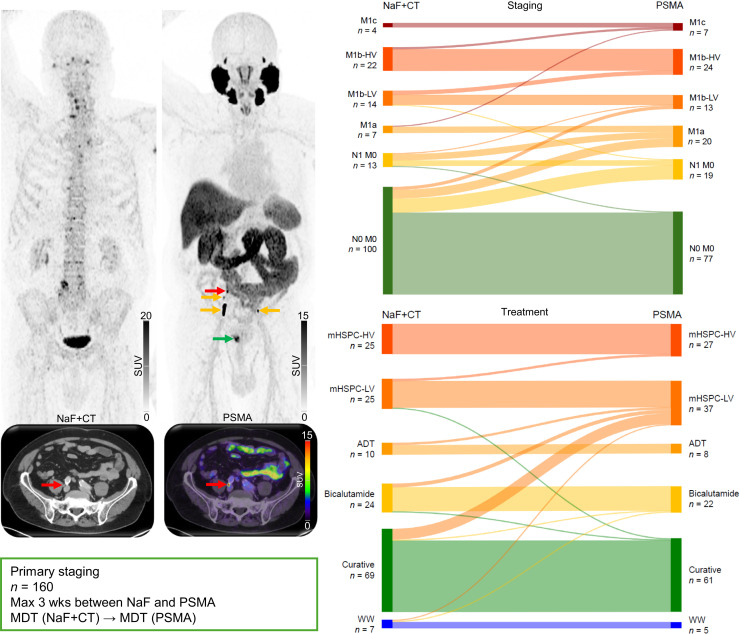

Diagnostic imaging of soft-tissue and bone metastases in patients with newly diagnosed prostate cancer (PC) is crucial for treatment planning. For decades, international guidelines have recommended structural imaging modalities, such as CT or MRI, to rule out lymph node and other soft-tissue metastases in primary staging (1,2). Lymph node size has been the primary criterion for determining the presence of metastatic involvement; however, CT has limited sensitivity and specificity for detecting lymph node involvement (3).

For decades, guidelines have recommended bone scintigraphy to evaluate potential skeletal involvement in patients with PC (1,2). More recently, ^18^F-NaF PET has gained approval as a more accurate alternative to bone scintigraphy (4,5). Both bone scintigraphy and ^18^F-NaF PET reflect osteoblastic bone formation, which is observed in, among other conditions, bone metastases (6,7). Thus, bone scintigraphy and ^18^F-NaF PET serve as indirect markers of metastatic involvement in PC.

In the past decade, radiolabeled prostate-specific membrane antigen (PSMA) ligands for PET imaging have emerged and been recognized for their potential in detecting soft-tissue and bone metastases in PC. These ligands bind to a glycoprotein on the cell surface that is upregulated in PC cells (8). Unlike the aforementioned conventional imaging modalities, PSMA PET/CT more directly reflects the presence of metastatic cells. Studies have demonstrated that PSMA PET/CT is superior to CT and bone scintigraphy in evaluating metastatic involvement during primary staging of PC (9). Consequently, the European Association of Urology has recommended PSMA PET/CT, when available, for primary staging in patients with high-risk or unfavorable intermediate-risk PC (10).

The use of the more sensitive PSMA PET/CT in clinical practice represents a paradigm shift in the staging of PC, often revealing more advanced disease and, as a result, altering treatment plans. Although large prospective studies have demonstrated the impact of PSMA PET/CT compared with bone scintigraphy and structural imaging (11,12), the clinical implications of replacing ^18^F-NaF PET/CT remain largely unexplored. To our knowledge, no large prospective studies have specifically addressed this substitution. The aim of this study was to evaluate the impact on primary staging and the consequences for treatment planning when implementing ^18^F-PSMA PET/CT in place of ^18^F-NaF PET/CT in patients with newly diagnosed PC.

## MATERIALS AND METHODS

This study complied with the Declaration of Helsinki. All patients gave written informed consent to participate. The study protocol was approved by the Regional Research Ethics Committee (approval H-20060829) and the Danish Data Protection Agency.

The study cohort comprised the same population as described in a previous study conducted by our group (13). Patients with histologically confirmed PC and categorized as high-risk according to the D’Amico criteria were included. All patients were referred from the Department of Urology for an ^18^F-NaF PET/CT scan as part of the standard diagnostic procedure at Copenhagen University Hospital Herlev and Gentofte. Study participants were enrolled between May 4, 2021, and February 20, 2024. The study-specific scan—^18^F-PSMA-1007 PET with contrast-enhanced CT, if tolerated—was performed within 3 wk of the ^18^F-NaF PET/CT. Unless specifically requested, the ^18^F-PSMA PET/CT results were not available for clinical decision-making. The results were made known only at the request of the clinical multidisciplinary team (cMDT) conferences, typically in cases of equivocal ^18^F-NaF PET/CT findings.

### Image Analysis

All ^18^F-NaF PET/CT and ^18^F-PSMA PET/CT scans were analyzed independently by 2 board-certified nuclear medicine physicians, each with extensive experience in interpreting these modalities. The CT scans derived from the ^18^F-PSMA PET/CT were evaluated independently by an experienced radiologist. All observers used version 8 of the *AJCC Cancer Staging System* to stage PC (14). Additionally, patients with bone metastases were categorized as having either low-volume disease (M1b–LV) or high-volume disease (M1b–HV), as defined by Sweeney et al. for the CHAARTED study (15).

Detailed information regarding specific criteria for the analysis of CT, ^18^F-NaF PET/CT, and ^18^F-PSMA PET/CT images is provided in the supplemental materials (available at http://jnm.snmjournals.org).

### Multidisciplinary Team Conferences

**FIGURE 1.**
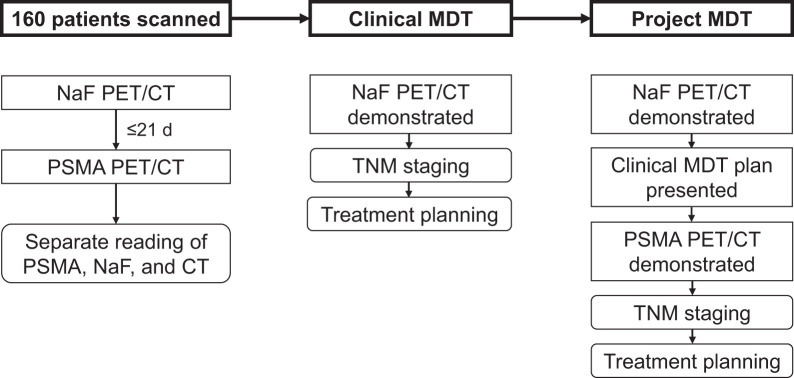
Flowchart summarizing image acquisition and interpretation, cMDT staging and treatment planning, and pMDT tentative staging and treatment planning.

Conventional imaging (CT and ^18^F-NaF PET) results were presented at cMDT conferences attended by urologists, an oncologist, a specialist in nuclear medicine, a radiologist, and a pathologist (Fig. 1). Clinical information—including clinical tumor stage, prostate-specific antigen level, International Society of Urological Pathology grade group, comorbidities, and performance status—was reviewed, and a treatment plan was determined.

The results and treatment plans determined at the cMDT conferences were subsequently presented at a project multidisciplinary team (pMDT) conference, attended by 2 experts in nuclear medicine, a urologist, and an oncologist. Treatments agreed upon at the cMDT were categorized as follows: watchful waiting, curative-intent treatment (all types), bicalutamide monotherapy, monotherapy with androgen deprivation therapy (ADT), low-volume metastatic hormone-sensitive PC (mHSPC-LV) treatment, and high-volume mHSPC (mHSPC-HV) treatment. Treatment for mHSPC-LV consisted of ADT combined with external beam radiotherapy to the primary tumor or an androgen receptor pathway inhibitor. Treatment for mHSPC-HV included ADT in combination with an androgen receptor pathway inhibitor or upfront docetaxel. For patients whose ^18^F-PSMA PET/CT had been revealed by request of the cMDT and used in clinical decision-making, the pMDT made tentative treatment plans based on the conventional imaging.

Subsequently, the results of the ^18^F-PSMA PET/CT were presented to the pMDT. The disease was restaged on the basis of the ^18^F-PSMA PET/CT findings, and the treatment plan was revised by the pMDT when relevant. This approach was also applied to patients who underwent low-dose CT alongside ^18^F-NaF PET.

### Statistical Analysis

Statistical analyses were performed using RStudio version 4.4.0. Patients’ clinical characteristics were reported using descriptive statistics, including mean, median, and range, as appropriate.

Wilcoxon signed-rank tests were performed to evaluate overall differences between groups before and after ^18^F-PSMA PET/CT. Staging categories were ranked from lowest to highest stage as follows: N0M0, N1M0, M1a, M1b-LV, M1b-HV, and M1c. Treatment categories were ranked as watchful waiting, curative-intent treatment, bicalutamide treatment, ADT as monotherapy, mHSPC-LV treatment, and mHSPC-HV treatment. Wilson method was used to calculate 95% CIs for proportions.

A *P* value of less than 0.05 was considered statistically significant. Sankey diagrams were created using SankeyMATIC.com.

## RESULTS

In total, 160 patients were enrolled in the study (Table 1). The mean interval between the conventional ^18^F-NaF PET/CT and the ^18^F-PSMA PET/CT scans was 10 d (range, 1–21 d).

**TABLE 1.** Study Population Characteristics

Characteristic	Value
Age (y)*	72 (54–88)
PSA (ng/mL)^†^	35 (2.3–7701)
PSA (ng/mL)	
<10	28 (18)
10–20	22 (14)
20.1–49.9	50 (31)
50–99.9	28 (18)
≥100	32 (20)
Clinical T-stage	
Tx	9 (6)
T1	12 (8)
T2a–T2b	13 (8)
T2c	21 (13)
T3	83 (52)
T4	22 (14)
ISUP grade group	
1–2	23 (14)
3	57 (36)
4–5	80 (50)

* Data represent mean and range.

† Data represent median and range.

PSA = prostate-specific antigen; ISUP = International Society of Urological Pathology.

Unless otherwise noted, data represent number and percentage.

### Change in Metastatic Stage

**FIGURE 2.**
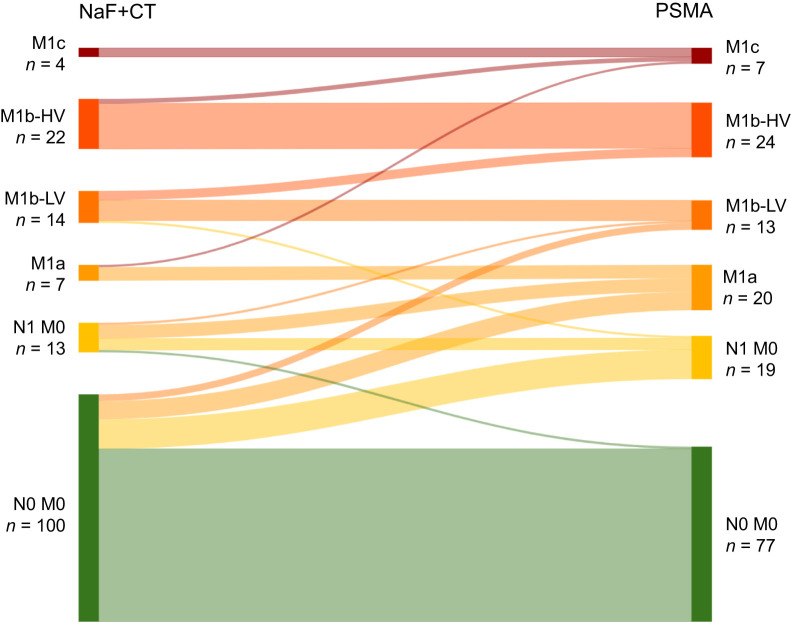
Sankey plot showing stage migration after ^18^F-PSMA PET/CT.

In total, 40 patients (25%; 95% CI, 19%–32%) experienced a change in metastatic stage after ^18^F-PSMA PET/CT results were added to the evaluation, compared with staging based on ^18^F-NaF PET and CT results alone (Fig. 2). Thirty-eight patients (24%; 95% CI, 18%–31%) were classified as having a more advanced stage and 2 patients (1%; 95% CI, 0%–4%) as having a less advanced stage (*P* < 0.001). The most notable stage migration occurred in relation to lymph node involvement. Thirteen patients (8%) without metastases on conventional ^18^F-NaF PET and CT were found to have regional lymph node metastases (N1M0), and 8 patients (5%) had metastases to distant lymph nodes (M1a). The number of patients with no metastatic involvement (N0M0) decreased from 100 (63%) to 77 (48%).

### Number of Lymph Nodes

**FIGURE 3.**
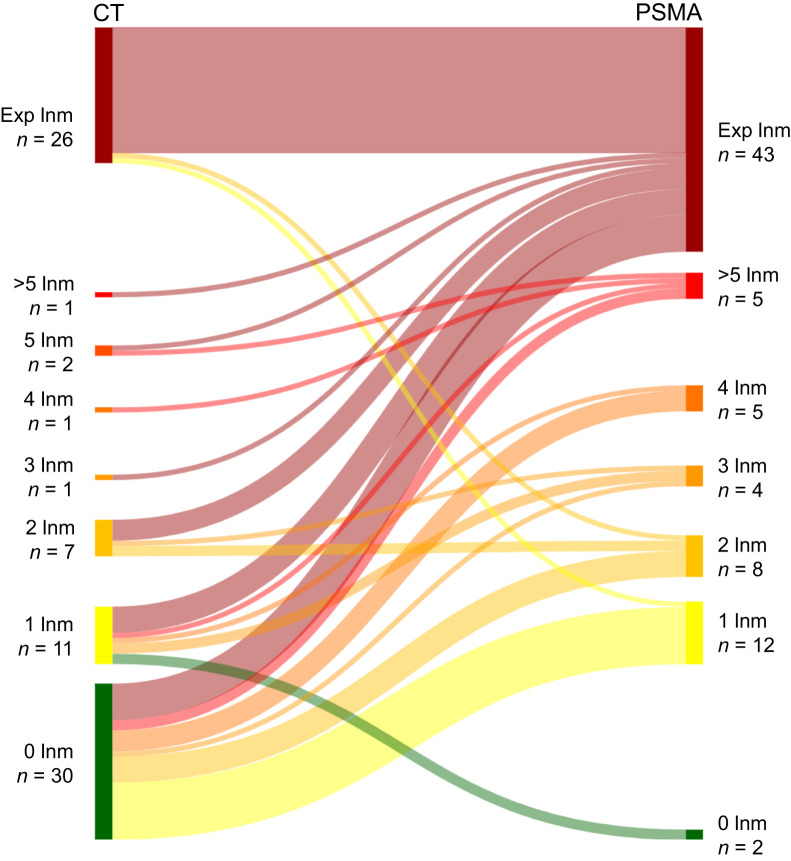
Sankey plot depicting 79 patients who had either intrapelvic lymph node metastases or extrapelvic lymph node metastases (any number) identified on CT or ^18^F-PSMA PET/CT. Exp = extrapelvic; lnm = lymph node metastases.

CT and ^18^F-PSMA PET/CT identified 81 patients (51%) without lymph node metastases on both imaging modalities. In the remaining 79 patients (49%), lymph node metastases were detected on one or both modalities (Fig. 3). Notably, 30 patients (19%) without lymph node metastases on CT had metastases detected on ^18^F-PSMA PET/CT, including 7 patients (4%) with extrapelvic lymph node involvement.

Across the entire cohort, the number of patients with extrapelvic lymph node disease increased from 26 (16%) on CT to 43 (27%) on ^18^F-PSMA PET/CT. Among 23 patients with pelvic-confined nodal disease on CT, 12 (52%) were found to have extrapelvic lymph node metastases on ^18^F-PSMA PET/CT. Furthermore, patients with only 1 or 2 intrapelvic lymph node metastases on CT exhibited a high risk of extrapelvic disease on ^18^F-PSMA PET/CT, observed in 45% and 57% of cases, respectively. Conversely, the disease stage of 2 patients was downgraded from N1 to N0 on the basis of ^18^F-PSMA PET/CT findings.

### Change in Treatment

**FIGURE 4.**
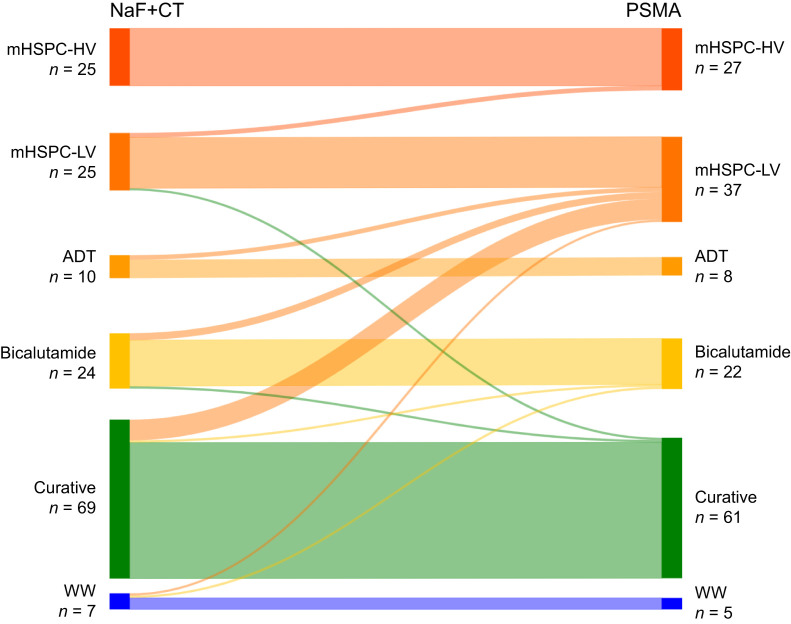
Sankey plot illustrating tentative treatment changes after ^18^F-PSMA results were provided. WW = watchful waiting.

Overall, treatment plans were modified for 21 (13%; 95% CI, 9%–19%) of the 160 patients based on ^18^F-PSMA PET/CT findings, with 19 patients (12%; 95% CI, 8%–18%) shifted to therapies targeting more advanced stages of disease (*P* = 0.001). Conversely, 2 patients (1%; 95% CI, 0%–4%) were reclassified as candidates for curative treatment (Fig. 4). The most notable impact on treatment plans was associated with the increased detection of lymph node involvement on ^18^F-PSMA PET/CT. Consequently, the largest increase was observed in the group planned for mHSPC-LV treatment, which rose from 25 patients (16%) to 37 patients (23%). Among the 69 patients initially considered for curative treatment, 8 (12%) were reclassified to receive noncurative treatment.

## DISCUSSION

In this study, we investigated the impact of ^18^F-PSMA PET/CT on staging and treatment planning in 160 patients with newly diagnosed PC and high-risk features. To our knowledge, this is the first prospective study to assess stage migration and the tentative clinical consequences if ^18^F-PSMA PET/CT replaces ^18^F-NaF PET and CT. We found that PC stage changed for 25% of patients, with 24% categorized as more advanced disease, mostly because of increased detection of lymph node metastases. In approximately 1 of 5 patients, ^18^F-PSMA PET/CT detected lymph node metastases that were not identified on CT.

Although stage migration occurred in 25% of all patients, the tentative treatment plan was altered in only 13%. Nevertheless, these findings illustrate the potential consequences of implementing a shift in daily clinical practice from conventional imaging modalities (such as ^18^F-NaF PET or bone scintigraphy and CT or MRI) to the more sensitive ^18^F-PSMA PET/CT, as recommended by international guidelines (10).

**FIGURE 5.**
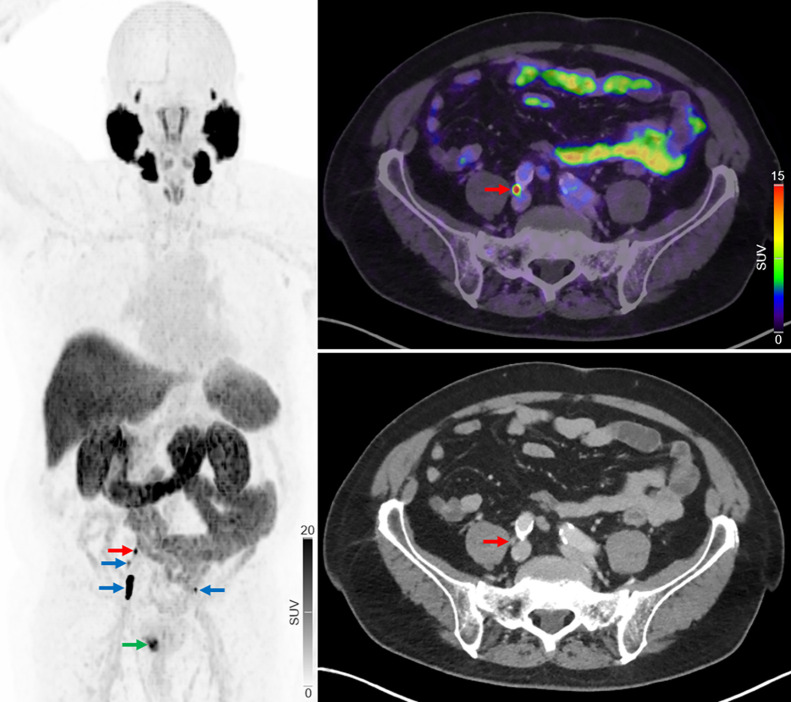
Primary staging of patient with high-risk PC. Initial ^18^F-NaF PET/CT showed no regional or distant metastases. Patient later underwent radical prostatectomy with extended pelvic lymph node dissection. In blinded project review, ^18^F-PSMA PET/CT revealed multiple intrapelvic lymph node metastases (blue arrows) and 1 lymph node metastasis near bifurcation of right common iliac artery (red arrows). Primary prostate lesion is also visible (green arrow). Three months postoperatively, patient’s PSA remained elevated (0.67 ng/mL), and bicalutamide therapy was started.

The staging categories with the greatest increase after ^18^F-PSMA PET/CT were N1M0 and M1a, with many of these patients initially classified as having disease in potentially curable stages (Fig. 5). Additionally, the number of detected lymph node metastases increased. Multiple studies, systematic reviews, and meta-analyses have demonstrated the superiority of PSMA PET/CT over CT or MRI for detecting lymph node metastases, using histopathology as the reference standard (9). However, despite the improved accuracy offered by PSMA PET/CT, the optimal management approach for patients whose stage is reclassified as more advanced with this imaging modality remains unclear (16), and the impact on long-term outcomes, such as progression-free and overall survival, is unknown.

To date, most studies have assessed the stage migration with and clinical impact of PSMA PET/CT compared with those of bone scintigraphy and structural imaging. In the proPSMA study, 27% of patients had treatment plans changed after the addition of ^68^Ga-PSMA PET/CT to conventional scans, consisting of bone scintigraphy and CT (11). A multicenter study by Roach et al. included a subgroup of 108 patients with primary PC (12). A change in management occurred in 21% of patients when ^68^Ga-PSMA was added to the imaging dataset.

In contrast to both aforementioned studies, our study found that treatment plans were altered in only 13% of patients. Several possible explanations may be considered. First, management plans were categorized differently across the studies. For instance, in our study, the curative category encompassed both radical prostatectomy and external beam radiation therapy, which were reported as separate categories in the proPSMA study (11). Second, the proPSMA study included only patients who were candidates for curative treatment, whereas our study enrolled all patients with newly diagnosed PC, including those with high-volume metastatic disease identified on ^18^F-NaF PET/CT. Finally, in our study, ^18^F-NaF PET/CT was used as the conventional imaging modality, whereas bone scintigraphy was used in the 2 aforementioned studies.

The lower sensitivity of bone scintigraphy was illustrated in a multicenter study by Bodar et al. (17). Nine of 70 patients (13%) had their disease stage increased from having no metastases on bone scintigraphy to low-volume metastatic bone disease, as defined in the CHAARTED study (15). Lymph nodes were not assessed. This finding aligns with the increased sensitivity of PSMA PET/CT in detecting bone metastases compared with bone scintigraphy (18). If the same subgroup analysis is applied to our study, only 4 (3%) of 160 patients would have their disease stage increased from having no bone metastases to low-volume bone metastases. Thus, the gap between M0 and low-volume metastatic bone disease appears narrower between ^18^F-NaF and ^18^F-PSMA PET/CT than between bone scintigraphy and ^18^F-PSMA PET/CT. Nevertheless, we previously demonstrated that ^18^F-PSMA PET/CT tends to detect more bone metastases than ^18^F-NaF PET/CT (13).

In a more recent retrospective multicenter study, Unterrainer et al. included a CHAARTED-like cohort of 67 patients with at least M1a disease on conventional imaging (bone scintigraphy, CT, or MRI) (19). This was compared with PSMA PET/CT performed within 100 d, and the patients were reclassified as having either high-volume metastatic disease, low-volume disease, or M0 disease. In total, the disease of 27 patients (40%) was restaged. Again, this contrasts with our study, where the disease of 6 of the 47 patients (13%) with at least M1a disease was restaged as M0, low-volume, or high-volume metastatic disease. In the study by Unterrainer et al., 16 (24%) of 69 patients had their disease stage lowered from M1 to M0 disease. The authors did not specify whether the discordant findings on conventional imaging were in lymph nodes, bones, or other organs, although a possible high false-positive rate on planar bone scintigraphy was mentioned (19). In our study, the disease of only 1 patient (1%) was downstaged to M0, providing evidence for the robustness of ^18^F-NaF PET/CT in detecting bone metastases (20,21).

Our study is not without limitations. The results of the ^18^F-NaF PET/CT and the resulting clinical treatment decisions were known to the pMDT when a tentative treatment plan based on ^18^F-PSMA PET/CT results was determined. Theoretically, the information provided by the ^18^F-NaF PET/CT could have influenced clinical decision-making on the basis of the ^18^F-PSMA PET/CT results.

In 22 cases, the ^18^F-PSMA PET/CT results were revealed because of clinical requests, primarily a result of equivocal findings on standard imaging. To mitigate potential bias, the pMDT initially made a tentative treatment decision solely on the basis of the ^18^F-NaF PET/CT results. Subsequently, the ^18^F-PSMA PET/CT findings were revealed, and a revised treatment plan was determined using the results of this modality. Since the pMDT determined treatment plans both before and after ^18^F-PSMA PET/CT, this approach may have facilitated a more uniform treatment reassessment in this subset of patients.

## CONCLUSION

To our knowledge, this study represents the largest prospective investigation to date examining changes in PC staging and clinical impact when ^18^F-PSMA PET/CT replaces ^18^F-NaF PET and CT for primary staging. Notably, 25% of patients were reclassified with respect to metastatic involvement, and 13% experienced a change in intended treatment. These findings are important, as PSMA PET/CT becomes increasingly integrated into international guidelines for primary staging.

## DISCLOSURE

No potential conflict of interest relevant to this article was reported.

## Notes

KEY POINTS **QUESTION:** What is the impact of replacing ^18^F-NaF PET/CT with ^18^F-PSMA PET/CT in primary staging of PC, as recommended in international guidelines? **PERTINENT FINDINGS:** In this prospective study of 160 patients with high-risk PC, 25% were reclassified in metastatic stage and 13% had a change in tentative treatment planning when ^18^F-PSMA PET/CT replaced ^18^F-NaF PET/CT. **IMPLICATIONS FOR PATIENT CARE:** The implementation of ^18^F-PSMA PET/CT upfront leads to stage migration and more advanced treatment strategies in a substantial proportion of patients.
